# Reciprocal Activating Crosstalk between c-Met and Caveolin 1 Promotes Invasive Phenotype in Hepatocellular Carcinoma

**DOI:** 10.1371/journal.pone.0105278

**Published:** 2014-08-22

**Authors:** Peyda Korhan, Esra Erdal, Emine Kandemiş, Murat Çokaklı, Deniz Nart, Funda Yılmaz, Alp Can, Neşe Atabey

**Affiliations:** 1 Department of Medical Biology, Dokuz Eylul University Medical School, Izmir, Turkey; 2 Department of Pathology, School of Medicine, Ege University, Izmir, Turkey; 3 Department of Histology and Embryology, School of Medicine, Ankara University, Ankara, Turkey; Xiangya Hospital of Central South University, China

## Abstract

c-Met, the receptor for Hepatocyte Growth Factor (HGF), overexpressed and deregulated in Hepatocellular Carcinoma (HCC). Caveolin 1 (CAV1), a plasma membrane protein that modulates signal transduction molecules, is also overexpressed in HCC. The aim of this study was to investigate biological and clinical significance of co-expression and activation of c-Met and CAV1 in HCC. We showed that c-Met and CAV1 were co-localized in HCC cells and HGF treatment increased this association. HGF-triggered c-Met activation caused a concurrent rise in both phosphorylation and expression of CAV1. Ectopic expression of CAV1 accelerated c-Met signaling, resulted in enhanced migration, invasion, and branching-morphogenesis. Silencing of CAV1 downregulated c-Met signaling, and decreased migratory/invasive capability of cells and attenuated branching morphogenesis. In addition, activation and co-localization of c-Met and CAV1 were elevated during hepatocarcinogenesis. In conclusion reciprocal activating crosstalk between c-Met and CAV1 promoted oncogenic signaling of c-Met contributed to the initiation and progression of HCC.

## Introduction

HCC is the sixth most common malignancy worldwide and the third most common cause of cancer related deaths [1], [2]. Although many advances have been made in the diagnosis and management of HCC, the prognosis of patients with HCC remains poor due to metastasis, recurrence and development of resistance to conventional chemotherapy and radiotherapy [1]–[6]. In recent years, improved knowledge of signaling pathways regulating HCC growth and progression has led to the identification of several novel molecular targets. One of the most promising signaling pathways for the molecular therapy of HCC appears to be the HGF/c-Met cascade [5]–[10].

c-Met was originally discovered as a proto-oncogene, expressed in epithelial cells and activated by its only known ligand, hepatocyte growth factor (HGF), which is secreted primarily by mesenchymal cells [11]. HGF, originally identified as a potent mitogen for hepatocytes, is involved in the development of a number of cellular phenotypes depending on a particular cell type and the microenvironment, including proliferation, morphogenesis and angiogenesis [11], [12]. Dysregulation of HGF/c-Met axis was reported to be linked to an unfavourable clinicopathological status, including high proliferation index, low degree differentiation, and vascular invasion and metastasis in a variety of cancer [9]–[16].

Experimental models of liver cancer and studies on human liver tissue samples have revealed that the outcome of HGF/c-Met activation would be both stimulation and inhibition of hepatocarcinogenesis [6], [8]–[10], [14]–[16]. Many studies have shown that overexpression of c-Met is correlated with a poor prognosis, including risk of tumor recurrence and short survival [6], [8], [15], [16]. However, no correlation has been found between HGF expression and histological grade or any other morphological features of HCC [17]–[19]. These results suggest that c-Met activation might occur through an HGF-independent mechanism during hepatocarcinogenesis [6],[8],[14]–[16]. Recent studies revealed the importance of signaling cross talk in resistance to receptor-targeted therapy [20],[21].

Lipid rafts play an important role in signaling crosstalk via bringing different proteins into proximity and thus promoting interactions between receptors and intracellular signaling proteins [22]. One of the major structural proteins of caveolae, CAV1, acts as a scaffolding protein by directly interacting with and modulating the activity of caveolae-localized signaling molecules [23]. Under physiological conditions, caveolae and CAV1 mediates endocytosis and transcytosis of molecules attached to the cell surface and organizes signaling proteins that participate in cell proliferation, adhesion and migration [24],[25]. The aberrant regulation and expression of CAV1 is involved in the pathogenesis of a variety of cancers. Depending on the tissue of origin CAV1 can be a tumor suppressor or initiator [25]. The increased expression of CAV1 in hepatocarcinogenesis has been shown to protect HCC cells from apoptosis and enhance the migration and invasion abilities of HCC cells [26]–[29]. On the other hand, the enhanced expression of CAV1 playing a tumor suppressive role in HCC has been also reported [30].

The co-localization of CAV1 and c-Met was reported in osteosarcomas and in human embryonic kidney cells, however, there is no study evaluating CAV1 and c-Met interaction and its biological consequences in HCC. Furthermore, co-localization between phosphorylated c-Met and phosphorylated CAV1 has not been determined.

In the present study, we report an association between CAV1 and c-Met modulated by HGF treatment, and the effects of this interaction on cellular motility, invasion and branching-morphogenesis. Additionally phosphorylated c-Met and phosphorylated CAV1 levels and their co-localization were determined in normal and cirrhotic liver and HCC tissues. These results add novel insights into the cooperativity of c-Met and CAV1 in HCC and the molecular mechanism behind the involvement of reciprocal activating crosstalk between c-Met and CAV1 in HCC progression.

## Materials and Methods

### Cell culture

Human HCC cell lines HuH-7, and SNU-449, were cultivated as described [31]. Authentication of cell lines was done by DNA profiling at the University of Colorado Cancer Center (UCCC) DNA Sequencing & Analysis Shared Resource (CO, USA) using Applied Biosystem's Identifiler kit (PN 4322288). Hepatocyte growth factor/scatter factor (HGF) was from R&D Systems (MN, USA). HGF (40 ng/mL) was used at specific time points after overnight starvation in DMEM with 2% FBS. For the inhibition of c-Met, SU11274 (Calbiochem 448101) was added to the cultures upon start of starvation. DMSO (Applichem) was used as vehicle for SU11274.

### Generation of Stable Cell Lines

HuH-7 cells were transfected with the plasmid pcDNA3.1/Myc-His (mock) and pcDNA3.1/-caveolin-1 (pCAV1). After transfection, cells were grown in selection medium containing 400 ug/mL geneticin (Life Technology 10131-027). Mock and CAV1 expressing cells both polyclonal and monoclonal were obtained and characterized by WB [26].

### Silencing of CAV1 Expression by Small-interfering RNA (siRNA) transfection

SNU-449 cells were transfected with 1 µM CAV1 (Accell Smart pool, E-003467-00, Dharmacon) (CAV1-siRNA) or non-targeting (Accell Smart pool, D-001 910-01, Dharmacon) (NT-siRNA) accell small interfering in Accell delivery media (Accell Dharmacon siRNA Delivery media, 303-604-9499), according to the manufacturer's instructions. A NT-siRNA was used as a control. 72 hours post-transfection, the cells were used for protein studies, migration, invasion and branching-morphogenesis assays.

### Motility and Invasion Assay

*In-vitro* motility and invasion assays were performed as described previously [26]. Briefly, NT- siRNA and CAV1 targeted siRNA pretreated SNU-449 and HuH-7-mock and HuH-7-pCAV1 cells were placed upper chambers. Lower wells of the inserts contained 2% FBS with/without HGF. After 24 h incubation at 37°C, the medium was removed and cells were fixed and stained with Diff Quick (Siemens Healthcare Diagnostics). Cells had traversed through the membrane were counted using a bright-field inverted microscope. Total cell numbers were counted for each chamber. Experiments were performed in at least triplicates. Bars represent fold differences in mean migrating or invading cell numbers. Fold differences were calculated by dividing the experimental results by the control results.

### Immunoprecipitation

Total cell lysates for IP and WB were prepared from SNU-449 cells with modified NP-40 buffer. Protein concentrations of samples were determined by the BCA assay following the manufacturer's instructions (Pierce, IL, USA). Total cell lysates were used to analyze the interaction between c-Met and CAV1 in SNU-449 cells. Samples were incubated with agarose conjugated anti-c-Met (sc-161 AC) antibody and bound proteins were analyzed by WB.

### Western Blotting

Total protein was prepared by using modified RIPA buffer as described previously [31]. Antibodies against phospho-Met (Y-1234/1235) (cell signaling (cs)-3129), phospho-Caveolin 1 (Y-14) (BD BioSciences 611339), phospho-p44/42-MAPK (Erk1/2) (Thr202/Tyr204) (cs-9101), p44/42-MAPK (Erk/21) (C-16) (sc-93), Caveolin 1 (sc-894), phospho-Src (Y-416) (cs-2101), Src (cs-2108), Calnexin (sc-11397) as described [31]. Equal loading and transfer were confirmed by repeat probing for calnexin (house-keeping gene). Band intensities were quantified as pixels by using ImageJ software (NIH).

### Immunofluorescence Microscopy

Cells seeded on BD Falcon chamber slides (BD BioSciences, 35118) were grown for 24 hours. After overnight starvation, HuH-7-mock and HuH-7-pCAV1 were treated with HGF. Cells were washed three times with ice cold PBS, then fixed with a mixture of 50% acetone and 50% methanol. Fixed cells were then blocked with 1% Bovine Serum Albumine (BSA) in PBS with 0.1% Triton X-100 (PBS-T) at room temperature (RT) and were incubated with primary antibodies against phospho-Met (Y-1234/1235) (cs-3129), c-Met (sc-161), phospho-caveolin-1 (Y-14) BD611339, caveolin-1 (sc-894). Double immunofluorescence with phospho-Met and phospho-CAV1 was performed on a Tissue Microarray (TMA) (US Biomax (BC03117)). sections incorporating a series of 48 cases of HCC, 22 liver cirrhosis, and 5 normal hepatic tissues, together with pathology diagnosis, grade, stage, TNM, HBV, type and history of hepatitis (yr) data. After deparaffinization step antigen retrieval using proteinase K was performed. from After secondary fluorochrome-conjugated antibodies (Alexa Fluor 488, invitrogen A-21206 and Alexa 555 invitrogen A-31570) treatment specimens were washed 6 times with PBS-T and slides were mounted with fluorescence mounting medium (Dako, S3023). Fluorophores from cell lines were visualized using Zeiss LSM 510 Meta confocal laser microscope (Jena, Germany) equipped with a Plan-Neofluar 40×/1.3NA oil objective at ambient temperature. Excitation and emission settings are as follows; for Alexa Flour 488: Ex: 488 nm/Em: 505–550 nm Band-pass filter; for Alexa Flour 555: Ex: 543 nm/Em: 560 nm Long-pass filter.DAPI was used to stain the cell nuclei. The detection parameters, such as laser intensity, amplifier offset and gain, and pinhole diameter, were fixed and kept at the same values for all specimens. Images were acquired using LSM510 ver.3.8 software and figures were generated using Adobe Photoshop. For co-localization analysis co-localization coefficients were calculated as c_1_ = pixels*_Ch_*_1,coloc_/pixels*_Ch_*_1,total_, where Ch1 and Ch2 are the first and second proteins to be detected. The given co-localization is expressed as the relative number of colocalizing pixels in channel 1 or channel 2, respectively, as compared to the total number of pixels above threshold. Threshold was set to 100 gray values. The entire TMA section was analyzed by acquisition of phospho-Met, phospho-CAV1 and DAPI signals by Immunofluorescence Microscopy using Olympus BX50 fluorescence microscope.

### Immunohistochemical procedure

phospho-Met (Y1234/1235) and phospho-CAV1 (Y14) expression profiles were analyzed by using immunoperoxidase staining in the serial liver sections. Expression of phospho-CAV1 and phospho-Met were examined in normal liver (n = 11), cirrhotic liver (n = 42), and HCC (n = 60) tissue samples. The clinicopathological characteristics of HCC patients are shown in Table S1. The study was approved by the Ethics Committee of Ege University Medical School. Written informed consents were obtained from patients before liver transplantation or liver biopsy sampling. All tissue samples were fixed in formalin and embedded in paraffin. Archival materials of the patients were reevaluated by a certified pathologist (DN) for the confirmation of the diagnosis and to choose the most appropriate tissue block for immunohistochemistry. The histopathological analyzes of all patients were carried out by the WHO histopathological classification of liver and intrahepatic bile ducts. Standard 5 µm tissue sections were taken on lysine-coated slides.

Sections were deparaffinized and then rehydrated. Immunostaining was performed using an automated immunohistochemical stainer according to the manufacturer's guidelines (IVIEW DAB, BenchMarkXP, and Ventana, USA). The antigen retriewal step was included in the automated programme. Endogenous peroxidase activity was blocked using 3% H_2_O_2_ for 4 minutes at 37°C. Endogenous avidin biotin activity was blocked by using the avidin-biotin block solution (DAKO, Denmark) and then primary antibodies anti-phospho-Met (Y-1234/1235) cs-3129, anti-phospho-Caveolin 1 (Y-14) BD-611339 were applied. The sections were stained with 3,3-diaminobenzidine tetrahydrocloride (DAB), a chromogen stain (brown in color), and counterstained with hematoxylin.

All staining were semi-quantitatively evaluated by certified pathologists (DN, FY) unaware of clinicopathological characteristics of the tumor. Expression of phospho-Met was defined as membranous and/or cytoplasmic when more than 10% of the hepatocytes stained positive for phospho-Met and phospho-CAV1. The extent of staining was scored as one positive (10 to 25% of cells were positively stained), two positive (26% to 50% of cells were positively stained) and three positive (more than 50% of the cells were positively stained). The intensity of phospho-Met and phospho-CAV1 immunostaining was semiquantitatively graded as follows: none (0), weak (+1), moderate (+2), and intense (+3).

### Branching-Morphogenesis Assays

HuH-7-mock, HuH-7-pCAV1, NT-siRNA or CAV1- siRNA treated SNU-449 cells were embedded in three-dimensional collagen I gels (BD 354236) as previously described [32]. Cultures were fixed with %4 paraformaldehyde after 21 days. For quantitation of the morphogenic response, 10 randomly selected areas per experimental condition in each of 4 independent cultures were photographed using 20× phase contrast objective using Olympus CKX41. All colonies on photographs were analyzed and scored for the ability to form branching tubules. The results were plotted as the average number of cysts able to undergo morphogenesis per culture per 100 [33].

### Statistical Analysis

Statistical analysis was performed using the GraphPad Prism and Statistical Package for Social Sciences 15.0 (SPSS Inc., Chicago, IL, USA). Statistical methods included Analysis of variance (ANOVA), Student's t-test, χ^2^-test. Correlation between two groups was assessed by Pearson's correlation analysis. p<0.05 was considered statistically significant.

## Results

### CAV1 associated with c-Met Tyrosine Kinase and this association increased by HGF stimulation

**Figure 1:**
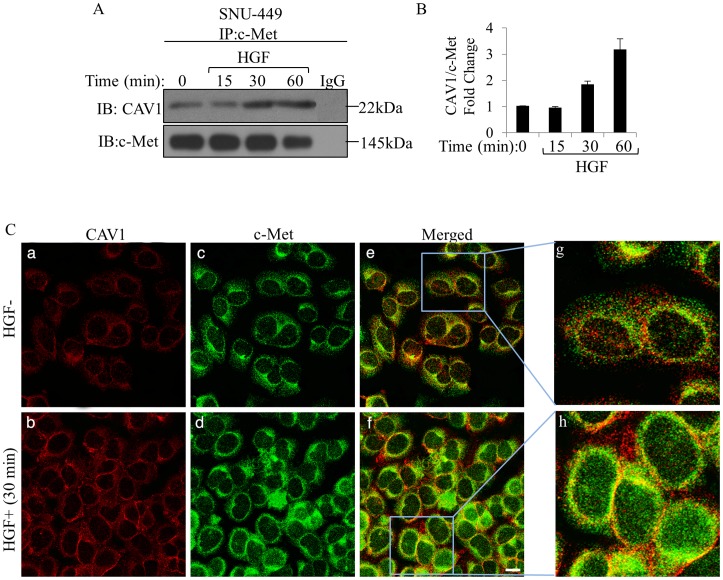
**A.** HGF induced association of CAV1 with c-Met. Serum starved SNU-449 cells were exposed to HGF for 15, 30 and 60 min. Whole cell lysates were immunoprecipitated with anti-c-Met antibody, resolved by SDS-PAGE and immunoblotted with antibody to CAV1 and c-Met. Anti-c-Met antibody was probed to the membrane as a loading control. There was no detectable c-Met and CAV1 band in immunoprecipitates prepared with IgG as an IP-control. **B.** The relative CAV1 intensities for the different treatments relative to the corresponding levels of c-Met were calculated. Different treatments relative to the present in the untreated control sample are compared in the bar graphs. Data were expressed as mean ± standard error (SE) of three independently experiments. **C.** Overnight starved SNU-449 cells were treated without (upper row)/with HGF (lower row) for 60 min. Representative image showing protein expression by double CM of CAV1 (red, Alexa 455) 1 (a, b), c-Met (green, Alexa 488) (c, d). Overlapping of red and green signals shown in yellow indicate the co-localization of the two proteins (e, f). Nuclei were stained with DAPI (blue). Blue boxed areas were enlarged in the insets to reveal the incidence of colocalization (g, h). Scale bar: 200 µm.

We used SNU-449 cells that are known to express both c-Met and CAV1 endogenously, to test for an association between c-Met and CAV1. The effect of HGF stimulation on this putative interaction was also evaluated. As shown in Fig. 1A and Fig. 1B modest levels of CAV1 were co-immunoprecipitated with c-Met from SNU-449 cells in the absence of HGF; and upon stimulation with HGF, a noticeable increase (≥3 fold) in the amount of co-immunoprecipitated proteins was observed (Fig. 1A, 1B). We obtained similar results when we analyzed Mahlavu cells, which have intrinsic CAV1 expression (see Fig. S1). Moreover, CM analysis showed that colocalization of CAV1 with c-Met (yellowish signal) was increased by HGF treatment (Fig. 1Cg, h). We also observed a rise in the fluorescent signal obtain from both CAV1 (Fig. 1Ca, b) and c-Met (Fig. 1Cc,d) after HGF induction, which indicated increased expression of CAV1 and c-Met in response to HGF (Fig. 1C).

### HGF stimulation increased both the phosphorylation and expression of CAV1

**Figure 2:**
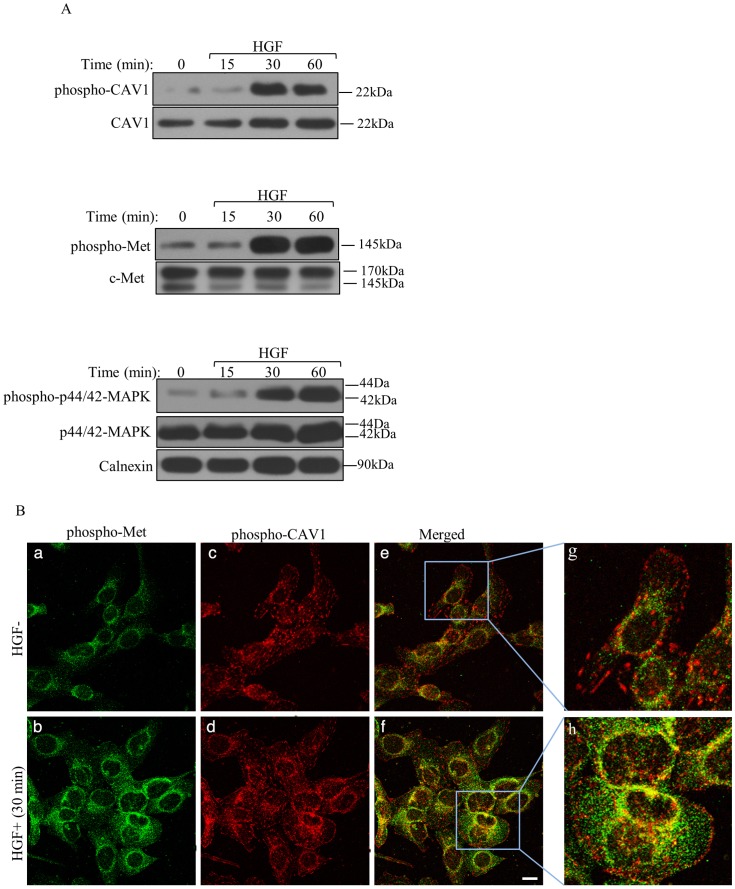
HGF mediated CAV1 activation. **A**. WB showing the expression of the indicated proteins in SNU-449 cells, overnight starved and treated with or without HGF at different time points. Calnexin was used as a loading control. **B**. Representative images for co-localization of phospho-CAV1 with phospho-Met increased by HGF treatment. Induced or non-induced SNU-449 cells by HGF for 60 min were assessed for c-Met (Y1234/1235) (green) (a, b) and CAV1 (Y14) (red) (c, d) phosphorylation by immune fluorescent staining. Subsequent images were merged (e and f) and overlapping of red and green signals were shown in yellow, indicating the co-localization of two proteins. A higher magnification view of the SNU-449 cells was also shown (see boxed areas) (g, h). Bar: 200 µm.

HGF stimulation increased the phosphorylation of CAV1 on tyrosine-14 (pY-14) in a time dependent manner (Fig. 2A). WB analysis supported the CM results shown in Fig. 1Ca,b that CAV1 expression was upregulated in response to HGF treatment (Fig. 2A). As expected, HGF induced phosphorylation of the tyrosine kinase domain at the autophosphorylation site (pY1234/1235) of c-Met (Fig. 2A). However, no change was observed in the expression level of c-Met. We also evaluated downstream signaling of HGF/c-Met cascade by analyzing activation statue of p42/44-MAPK (Erk1/2), which is a well-known component of c-Met signaling pathway [33]. WB analysis demonstrated increased phosphorylation of p44/42 MAPK (Erk1/2) in HGF treated cells compared to untreated cells (Fig. 2A). Expression of p44/42-MAPK was also elevated by HGF treatment. As we previously reported, we observed similar results when we analyzed Mahlavu cells [33].

We also evaluated the cellular distribution of phospho-Met and phospho-CAV1 in SNU-449 cells by CM (Fig. 2B). Consistent with the data obtained from WB analysis, confocal z-axis image stacks revealed an increase in the phosphorylation of c-Met (Fig. 2B a,b) and CAV1 (Fig. 2B c,d) in response to HGF. Furthermore co-localization between phospho-Met and phospho-CAV1 was enhanced by HGF, compared to basal conditions (Fig. 2B e,f,g,h). These data implied that HGF triggered the phosphorylation of CAV1 on tyrosine 14 and enhanced co-localization of CAV1 with c-Met. Moreover, we observed that co-localization signals obtained from phospho-Met and phospho-CAV1 were accumulated predominantly in the perinuclear area (Fig. 2B g, h).

### Inhibition of c-Met activation abolished phosphorylation of CAV1 on Tyrosine 14

**Figure 3:**
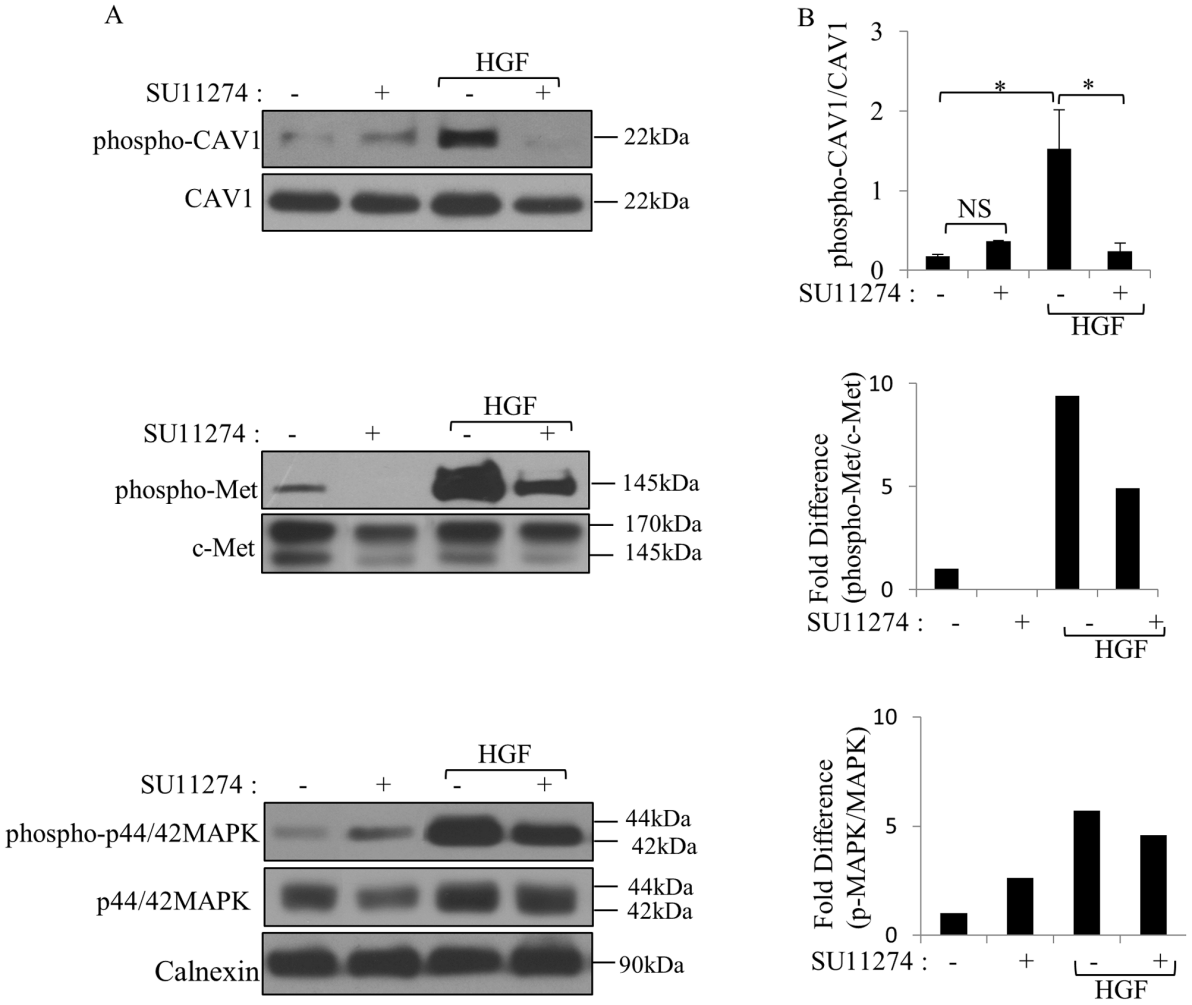
SU11274 inhibited c-Met and downstream signaling. **A.** After overnight starvation and SU11274 pretreatment, SNU-449 cells were stimulated with HGF for 15, 30, 60 min or left un-stimulated. Lysates were immunoblotted with indicated antibodies. Calnexin was used as loading control. **B.** Band intensities of phospho-CAV1 were quantified by densitometry and normalized to CAV1 (n = 3) (*p<0,05, NS: not significant). The relative phospho-Met/c-Met and p-MAPK/MAPK for the different treatments relative to the levels present in corresponding untreated control samples are compared in the bar graphs (n = 3).

To confirm CAV1 phosphorylation was mediated by c-Met activation, we used c-Met receptor kinase inhibitor, SU11274, to block c-Met activation and investigated the effect of c-Met inhibition on CAV1 phosphorylation. HGF-mediated CAV1 phosphorylation was decreased by SU11274 treatment, parallel to the decrease observed with c-Met phosphorylation (Fig. 3). Overnight treatment of SNU-449 cells with SU11274 remarkably decreased HGF-induced both c-Met and MAPK phosphorylation (Fig. 3). These results indicate that CAV1 phosphorylation is dependent on c-Met activation.

### Ectopic expression of CAV1 accelerated c-Met signal transduction pathway

**Figure 4:**
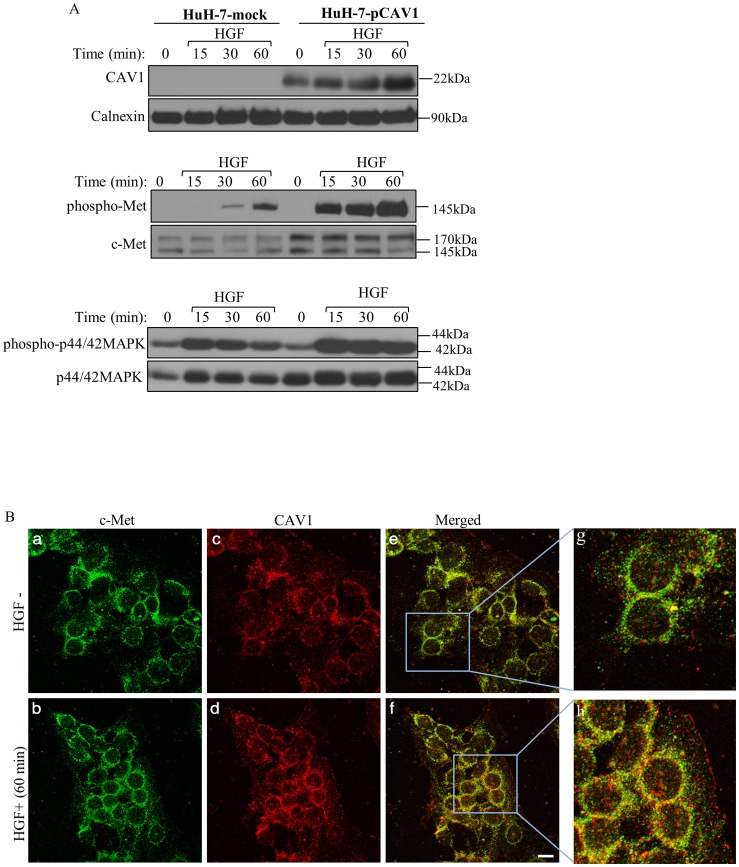
Ectopic expression of CAV1 enhanced HGF-mediated c-Met activation. **A.** Huh-7-mock and HuH-7-pCAV1 cells were starved for overnight prior to stimulation with HGF for 15, 30 and 60 min. Expression of phosphorylated and total c-Met and MAPK were examined by WB. Overexpression of CAV1 was also verified by WB. Calnexin is shown as a loading control. **B.** After 60 min treatment with HuH-7-mock, cells stably expressing CAV1 were fixed and stained for c-Met (green) (a, b) and CAV1 (red) (c, d), colocalization signal (yellow) (e, f). Representative fluorescence micrographs were obtained by CM. Higher magnification images were presented to reveal the incidence of colocalization (g, h). Bar: 200 µm.

Findings obtained using SNU-449 cells suggested that c-Met positively regulated CAV1 activity. To examine the effect of CAV1 on the c-Met signaling pathway, we first introduced CAV1 gene into CAV1-negative and c-Met-positive cell lines, HuH-7 and Hep G2, by stable cloning to establish CAV1 overexpressing stable clones. Then, we treated HuH-7-pCAV1 and HuH-7-mock clones with HGF and subjected to WB analysis. We determined that HGF treatment increased CAV1 expression in CAV1 clones (Fig. 4A). Upon HGF binding, HGF induced c-Met activation, which was higher and was sustained in CAV1 clones compared to mocks over the 24-h time course of the experiments (Fig. 4A, and Fig. S2). Moreover, c-Met expression was higher in HuH-7-pCAV1 cells compared to the HuH-7-mock for all the time points examined (Fig. 4A, Fig. S2). Parallel to these findings, both the expressions and phosphorylation of p44/42-MAPK were higher in CAV1 clones compared to mocks at all the time points examined (Fig. 4A and Fig. S2). We obtained similar results with Hep G2–pCAV1 clones as well (data not shown). Similar to the result from SNU-449 cells, signals obtained from c-Met (Fig. 4B a, b) overlapped with the signal acquired from CAV1 (Fig. 4B c,d) at multiple points, which was amplified by HGF induction (Fig. 4B e,f,g,h).

### The overexpression of CAV1 increased HGF-induced cell migration, invasion and branching-morphogenesis

**Figure 5:**
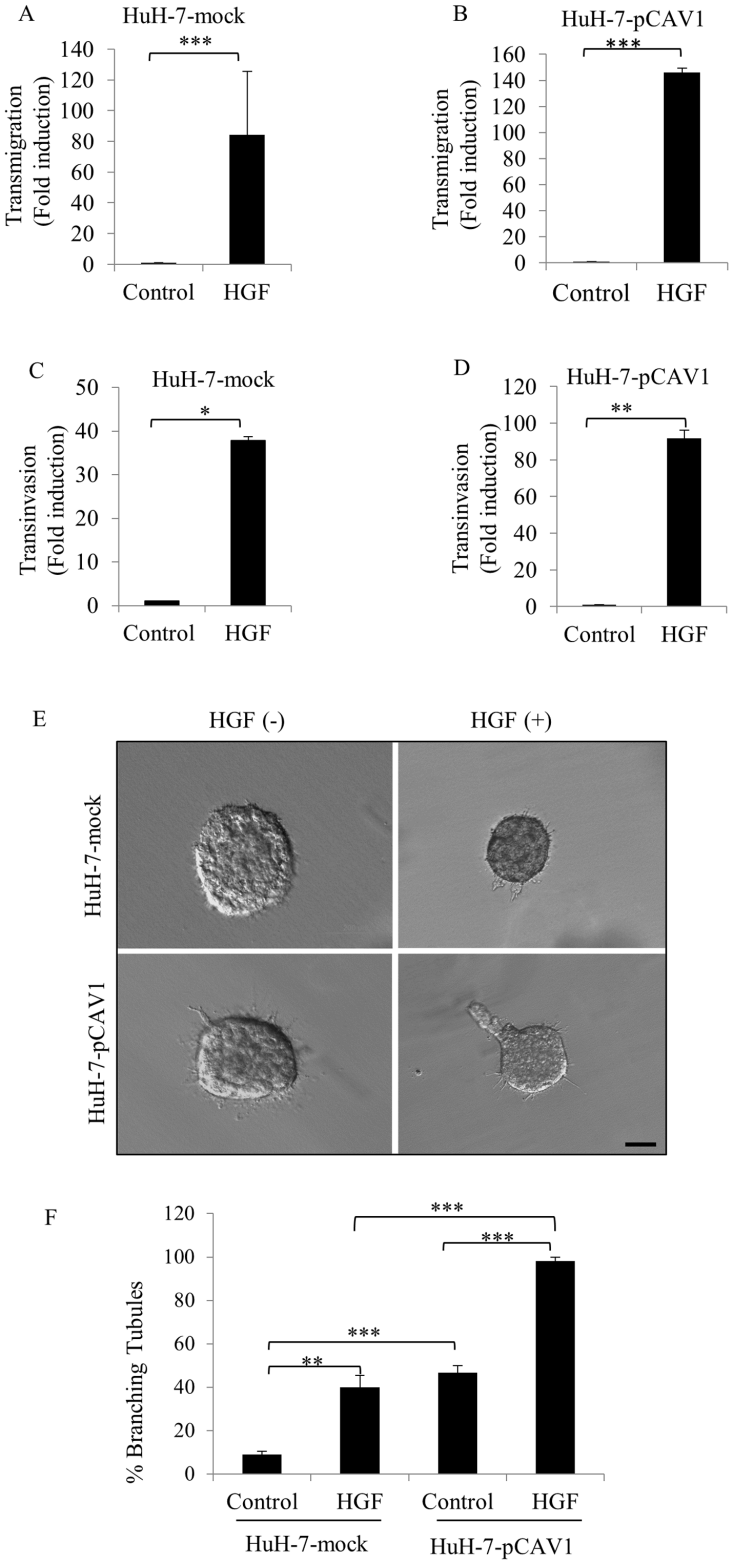
Ectopic expression of CAV1 enhanced c-Met signaling to drive cellular motility and invasion and branching morphogenesis in response to HGF stimulation. Cells transfected with mock or CAV1 plasmid were treated with or without HGF and then tested in migration and invasion assays. The cells that migrated and invaded through membrane were stained, counted under a light microscope at 25× magnification. The results are representative of three independent experiments, done in quadruplicate. Fold change were calculated by data from non-induced conditions. Bars represent fold change in mean number ± S.E. of migrating and invading cells; **A.** HuH-7-mock, **B.** HuH-pCAV1 and invading cells; **C.** HuH-7-mock, **D.** HuH-7- pCAV1 Student's t-test was used in the comparison of the means of non-induced and HGF-induced conditions. **E.** Branching-morphogenesis assay performed by HuH-7-mock and HuH-7-pCAV1 cells in DMEM supplemented with or without HGF. **F.** Columns shows mean number ± S.E derived from three separate experiments done in quadruplicate. ANOVA was used in comparison of groups. (***p<0,0001, **p<0,001, *p<0,05; Bar: 200 µm.).

HGF-mediated c-Met activation generates a variety of cellular responses, including motility, invasion and branching morphogenesis. When we tested the role of CAV1 in HGF/c-Met induced motility and invasion we observed that HGF treatment caused approximately an 84-fold increase in the motility of HuH-7 mock cells compared to non-induced ones (p<0.0001) (Fig. 5A). Remarkably, HGF treatment induced a ≥146-fold increase in the migration capacity of HuH-7-pCAV1 cells, compared to un-treated cells (p<0.0001) (Fig. 5B). Similarly, invasion assays showed that HGF treatment elevated Matrigel invasion capacity of HuH-7-mock cells about ≥37-fold compared to the non-stimulated mocks, (p<0.05) (Fig. 5C). Furthermore, HGF promoted invasion of HuH-7-pCAV1 cells to ≥91-fold (p<0.001) compared to non-treated HuH-7-pCAV1 clones (Fig. 5D).

We also tested the ability of these clones to undergo tubular morphogenesis in collagen gel. At basal conditions, mock-transfected cells formed mainly cysts, whereas cells expressing CAV1 formed branches and tubules. Representative clones are shown in Fig. 5E. Quantitation of the response of mock and CAV1 clones revealed that 9% of mock and 47% of CAV1 of the performed cysts were able to undergo morphological changes (Fig. 5F). As expected, in the presence of HGF, both mock and CAV1 clones formed branches and tubules, whereas HuH-7-pCAV1 cells formed longer and more complex branching tubules compared to mocks (Fig. 5E, 5F). Under HGF stimulation, 40% of control cells formed branching tubules, whereas almost all CAV1 transfectants (98%) formed tubules (Fig. 5F). Taken together, these observations suggest that the overexpression of CAV1 enhanced HGF-mediated branching-morphogenesis.

### Silencing of CAV1 expression suppressed c-Met activation

**Figure 6:**
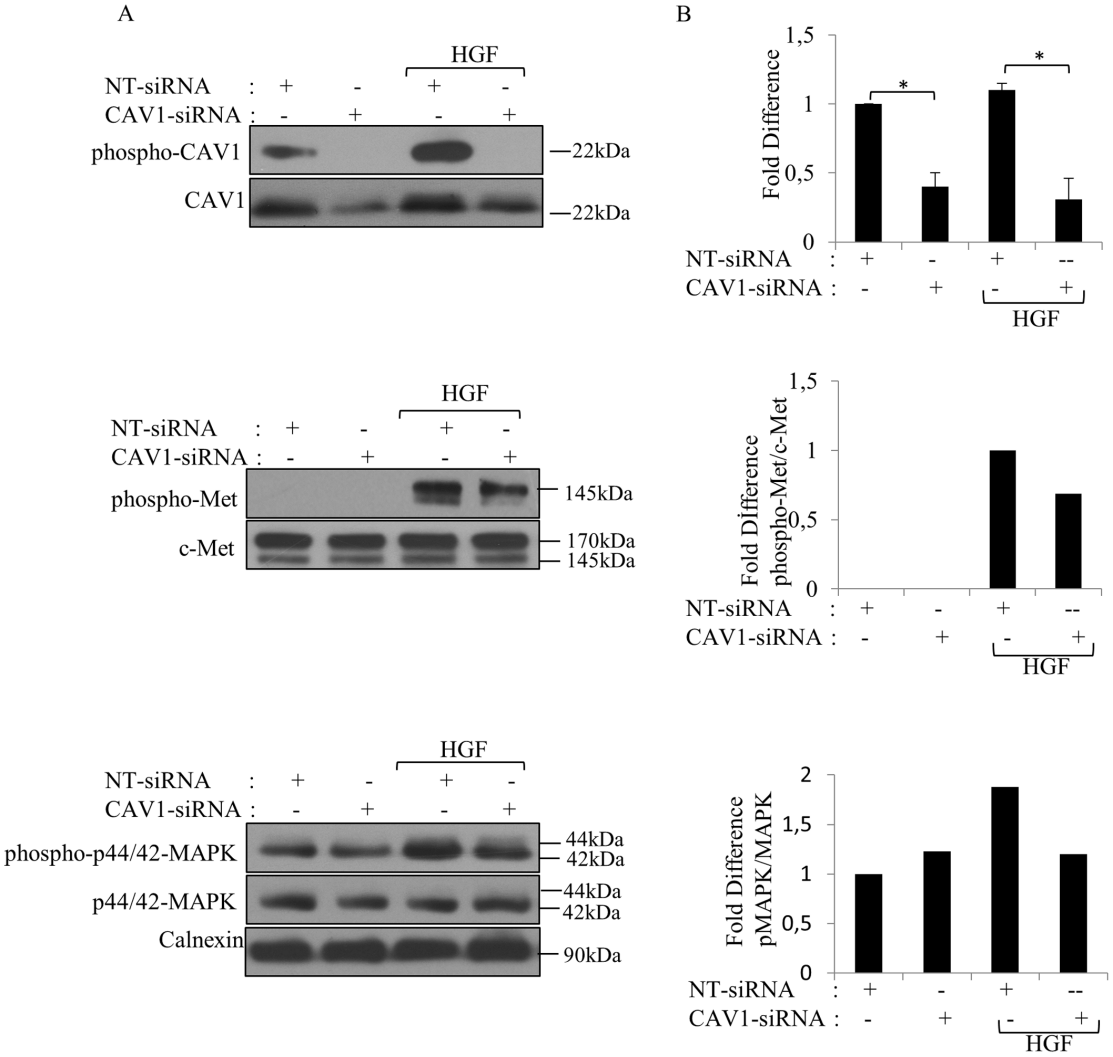
CAV1 knockdown inhibited HGF evoked activation of c-Met signaling pathway. **A.** SNU-449 cells transfected with CAV1-siRNA or NT-siRNA for 72 h, stimulated with HGF for 30 min were subjected to WB analysis with indicated antibodies. Calnexin was used as a loading control. **B.** The graph depicting results from densitometry quantitation of CAV1 band which was normalized to calnexin. Error bars represent S.E. (n = 5). Band intensities of phospho-Met and p-MAPK were quantified by densitometry and normalized to c-Met and MAPK, respectively (n = 3). Values from NT-siRNA were set at 1. ANOVA was used in comparison of groups. (*p<0,05, NS: not significant).

Based on the observation that c-Met activation accelerated both the expression and phosphorylation of CAV1 protein, we hypothesized that the silencing of CAV1 had an effect on the c-Met transactivation pathway. To test this, we used siRNA to deplete CAV1 expression in SNU-449 cells and analyzed c-Met activation. We determined that siRNA treatment abolished both basal and HGF induced CAV1 phosphorylation (Fig. 6A). As shown in Fig. 6B, treatment with CAV1 siRNA decreased CAV1 protein expression significantly in SNU-449 cells by approximately ≥60% compared with the cells transfected with NT-siRNA (p<0.05). Furthermore HGF increased CAV1 expression, CAV1-siRNA was sufficient to reduce CAV1 levels by ≥80% compared to NT-siRNA treated group under HGF stimulation (p<0.05) (Fig. 6A). c-Met activation was diminished by CAV1 depletion compared to the control group and this result reflected on MAPK activation as well. These data suggest that CAV1 has a positive regulatory role on c-Met signaling.

### The silencing of CAV1 reduced migration, invasion, and branching morphogenesis

**Figure 7:**
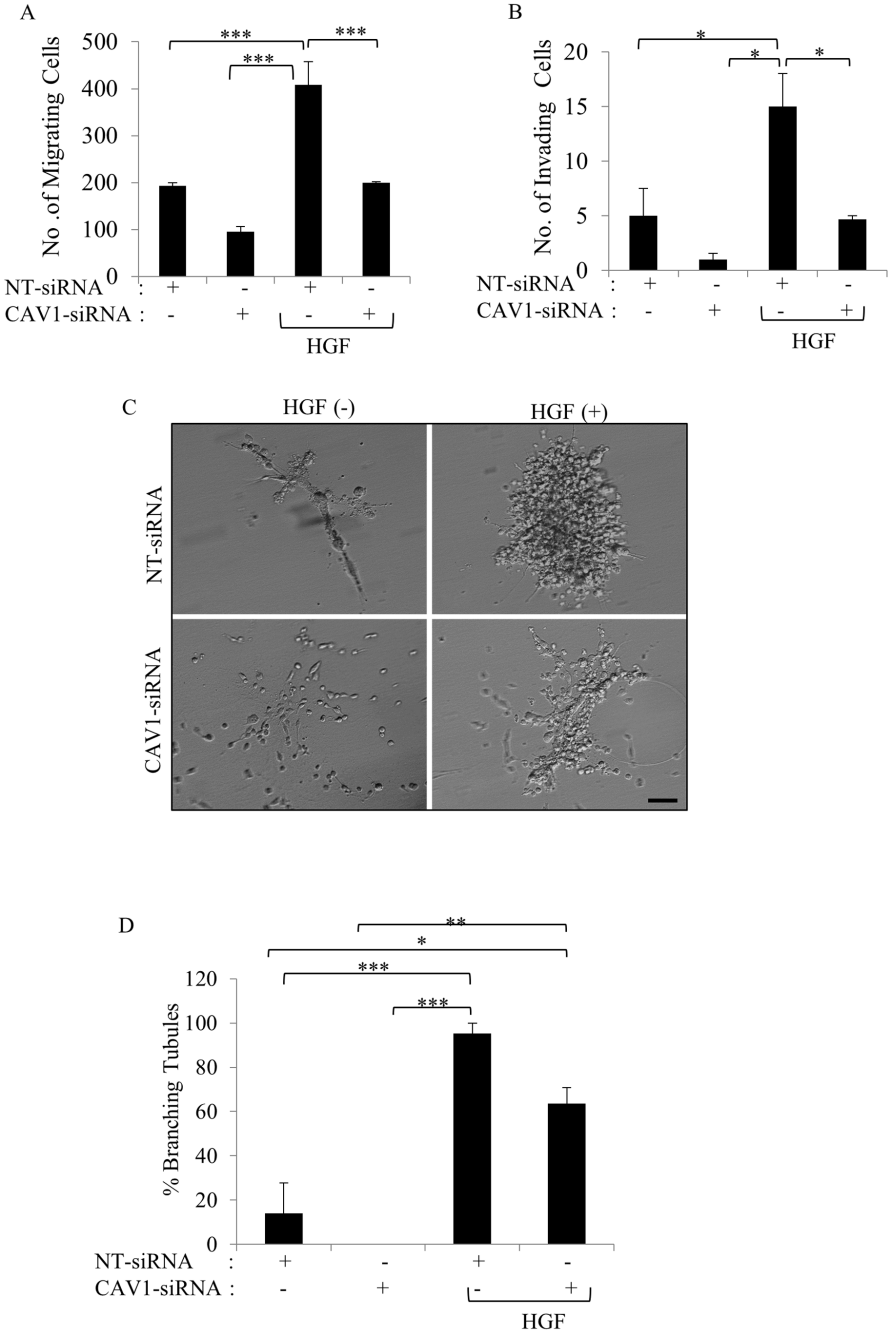
Silencing of CAV1 inhibited the migration and invasion of SNU-449 cells and decreased the formation of complex branching tubular structures. NT- and CAV1 siRNA transfected SNU-449 cells were seeded in the upper chamber of Boyden chambers. Medium with or without HGF was added to the lower chamber. After 24 h incubation, migrated and invaded. Cells were counted. Bars represent the mean three independent experiment ± S.E. of **A.** migrating or **B.** invading cell number. **C.** SNU-449 cells were transiently transfected with CAV1 or NT-siRNA were grown in collagen I gel for 21 days. HGF was added as indicated. Branching-morphogenesis responses were followed up to 21 days and tubule formation was evaluated. **D.** Images show a representative experiment that had been performed in quadruplicate for each clone of each group. All data are represented as fold change± S.E. compared with control cells. Photographs were taken at 21 st day by Phase-contrast microscopy. ANOVA was used in comparison of groups, (***p<0,0001, **p<0,001, *p<0,05, Bar: 200 µm).

As CAV1 knockdown suppressed the activation of c-Met signaling pathway, we next asked whether CAV1 silencing inhibits cell migration, invasion and branching morphogenesis in response to HGF. As shown in Fig. 7A CAV1 silencing reduced motility by approximately half compared to control group. However, this result was not statistically significant (p>0.05). HGF treatment significantly increased migration compared to all groups tested (p<0.001). CAV1-siRNA-treated cells exhibited lower migratory capability compared to NT-siRNA treated group under HGF induction (p<0.001) (Fig. 7A). In the invasion assay, the number of HGF-treated cells to invade through filter-coated with Matrigel was significantly higher compared to all groups tested (p<0.05) (Fig. 7B). Although CAV1 knockdown decreased the invasion potential of cells compared to the control group, no statistically significant reduction was observed (Fig. 7B). Consistent with the observation in the motility assay, CAV1 silencing reduced HGF-induced cell invasion significantly (p<0.05) (Fig. 7B). These results indicate that the silencing of CAV1 played an inhibitory effect on the migration and invasion of SNU-449 cells. We next examined the ability of NT-siRNAtransfected and CAV1-siRNA-transfected SNU-449 cells to form branching-morphogenesis in collagen gel in the presence or absence HGF. While approximately 14% of the control cells formed branching tubules, CAV1-silent cells failed to develop branching tubules (p>0.05) (Fig. 7C, D). As expected, in the control group HGF enhanced the formation of branching tubules by more than 95% (p<0.0001). However; CAV1-silent cells exhibited lower ability to form tubules (64%) and produce branches shorter tubules compared to the control cells under HGF induction (p>0.05) (Fig. 7C, D). These results suggest that the silencing of CAV1 reduced the ability of SNU-449 cells to form tubules.

### phospho-CAV1 and phospho-Met expressions and co-localization higher in HCC tissues than normal and cirrhotic liver samples

**Figure 8:**
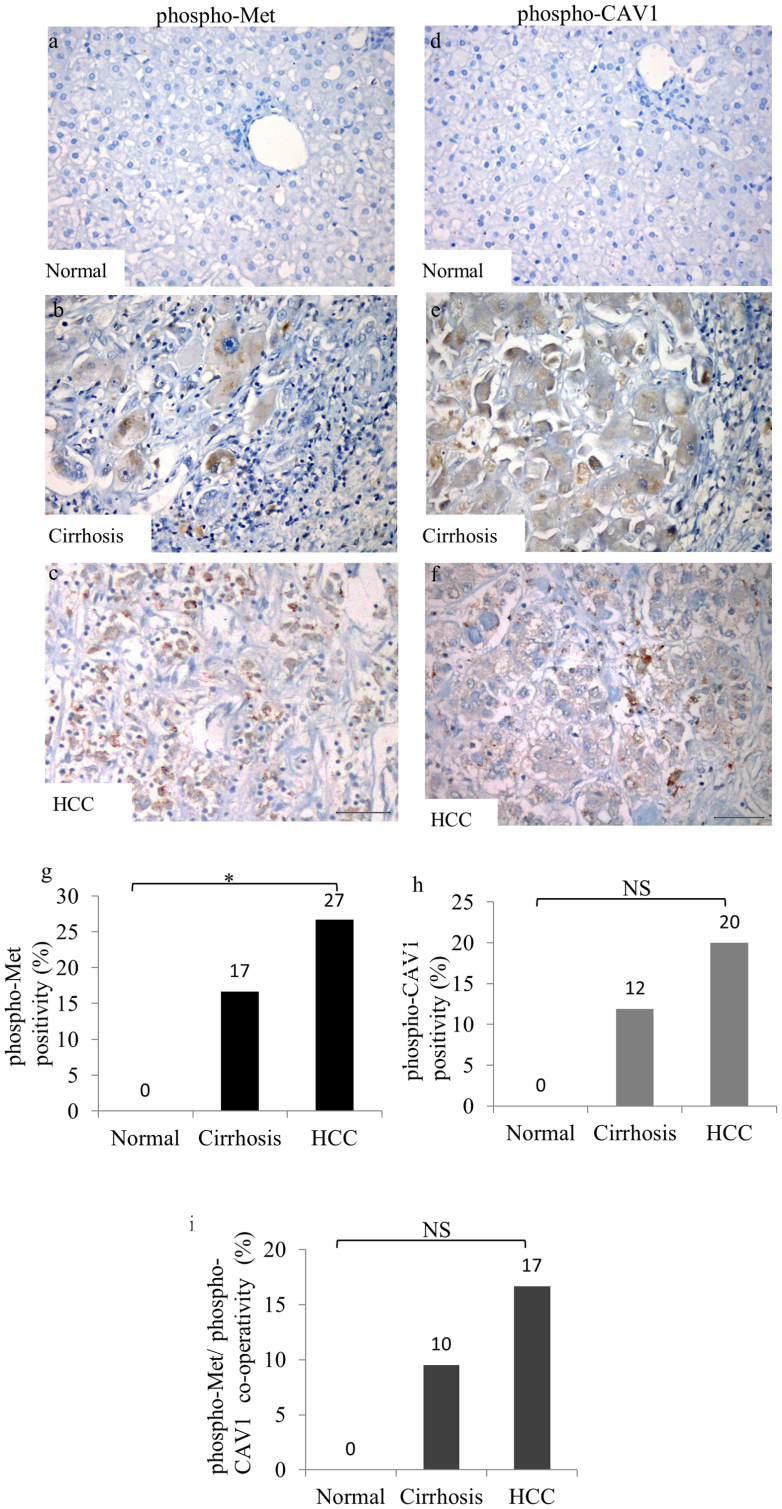
Immunohistochemical assesment of phospho-Met and phospho-CAV1 expressions in normal, cirrhotic and HCC tissues. Negative phosphor-Met expression in normal hepatocytes (a), cirrhotic liver tissue showed weak, phospho-Met staining (b). HCC displayed intense phospho-Met staining. Each column represents histologically classified liver tissues (normal liver, cirrhotic liver, HCC) with the height representing the ratio of positive staining for phospho-Met (d). Negative phospho-CAV1 expression in normal liver tissue (e), diffuse phospho-CAV1 staining in hepatocytes in the cirrhotic liver tissue (g), HCC displayed strong phospho-CAV1 staining (h). Comparison of the ratios of positive staining for phospho-CAV1 in normal liver, cirrhotic liver, and HCC tissues. Each column represents the immunoreactivity of both phospho-Met and phospho-CAV1 in the same liver tissue samples (i). Trend in χ^2^-test was performed to determine the trend between groups (*p<0,05, NS: not significant, Bar = 200 µm).

**Figure 9:**
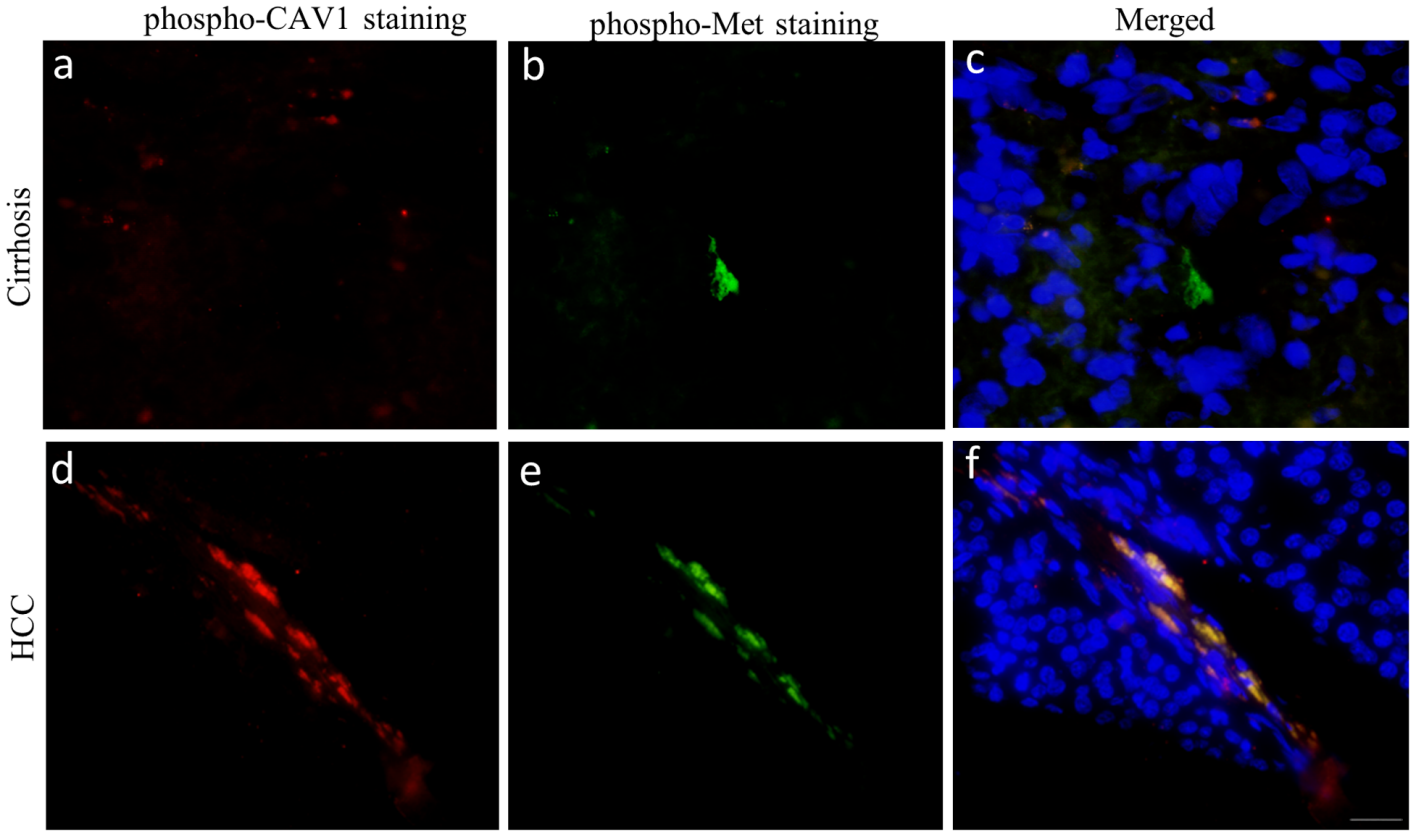
Immunofluorescence analysis of co-localization between phospho- CAV1 and phospho- Met in cirrhotic and HCC tissues. Image representing phospho-CAV1 (red) (a) and phospho-Met (green) (b) and no colocalization (yellow) (c) in cirrhotic tissue section. Representative image showing phospho-CAV1 (red) (d) and phospho-Met (green)(e) and colocalization (yellow) (f) in HCC tissue section. Nuclei were stained with DAPI (blue). (Bar = 200 µm).

Since we observed reciprocal regulatory functions of CAV1 on c-Met and vice versa in cell culture studies, we next analyzed their correlation in liver tissues. No demonstrable phospho-Met (Fig. 8a) and phospho-CAV1 (Fig. 8d) staining were observed in normal liver tissues. On the other hand, positive staining for phospho-Met (Fig. 8b) and phospho-CAV1 (Fig. 8e) were observed in 17% (7/42) and 12% (5/42) of cirrhotic liver tissues sections, respectively (Fig. 8g, h). In cirrhotic livers, weak phospho-Met and phospho-CAV1 staining were determined in hepatocytes, bile duct cells, and lymphocytes. In HCC tissues, 27% (16/60) positive staining for phospho-Met (Fig. 8c, g) and 20% (12/60) positive staining for phospho-CAV1 (Fig. 8f, h) were observed. Positive staining was defined as membranous and/or cytoplasmic in reactive tumor cells for phospho-Met, and phospho-CAV1. The levels of phospho-CAV1 and phospho-Met expression were semiquantitatively graded as 0 (less than 10% of the cells), 1+ (positivity between 10%–50% of cells), and 2+ (more than 50% of cells). The intensity of phospho-CAV1 and phospho-Met expressions was semiquantitatively graded as 0, 1 or 2. There was a significant gradual increase in phospho-Met staining through normal, cirrhotic and HCC tissues (p<0.05). Although there was an increase in phospho-CAV1 staining towards normal to cirrhosis and to HCC, this result was not statistically significant (p>0.05). The immunoreactivity of both phospho-Met and phospho-CAV1 in the same liver tissue sample showed a similar staining pattern that was absent in normal liver tissues, present in cirrhosis and increased in HCC compared to normal and cirrhotic tissues (Fig. 8i). Pearson's Correlation test was performed in two matched groups: *i)* cirrhotic tissues staining with phospho-Met and phospho-CAV1; *ii)* HCC tissues staining with phospho-Met and phospho-CAV1 analysis revealed that phospho-CAV1 expression was correlated positively with phospho-Met in cirrhosis (r = 0.70, p<0.0001) and HCC (r = 0.73, p<0.0001). We next investigated the association between phospho-Met and phospho-CAV1 in normal, cirrhotic and HCC liver sections. Consistent with the data obtained from IHC, no phospho-Met and phospho-CAV1 expression were detected by IF microscopy (data not shown). Interestingly, while no co-localization was detected between phospho-CAV1 and phospho-Met (Fig. 9b) in cirrhotic liver tissues (Fig. 9c), a significant co-localization between phospho-CAV1 (Fig. 9d) and phospho-Met (Fig. 9e) was detected in HCC (Fig. 9f).

**Figure 10:**
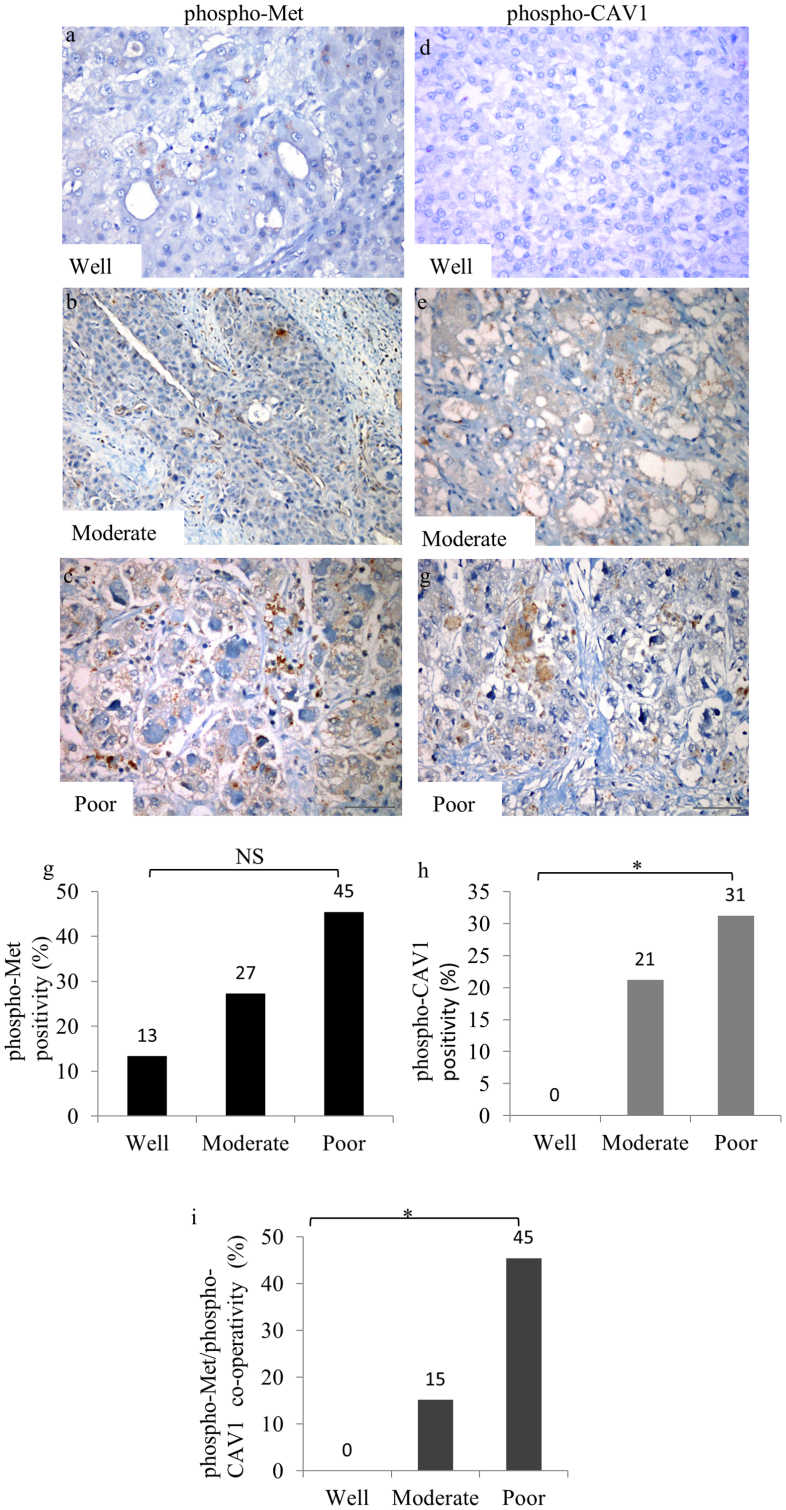
Immunohistochemical staining in well-, moderate-, and poorly differentiated HCC tissues revealed an increasing trend towards well to poor differentiation. Representative images shows Serial sections were stained with anti-phospho-Met (a, b, c) and anti-phospho-CAV1(d, e, f) antibodies. Each column represents the ratio of positive staining for phospho-Met (g) and phospho-CAV1 (h), co-expression of phospho-Met and phospho-CAV1 (i) in well-, moderate-, and poorly-differentiated HCC. Trend in χ^2^-test was performed to determine the trend between groups (*p<0,05, NS: not significant, Bar = 200 µm).

We further analyzed the expression of phospho-Met and phospho-CAV1 with respect to histological differentiation. The levels of phospho-Met positive staining were 13% in well-, 27% in moderate- and 45% in poor-differentiated HCC tissues (Figs. 10a–c, 10g). We performed χ^2^-test to determine the trend between groups. No significant differences were found between groups for phospho-Met staining (p>0.05). phospho-CAV1 positive HCC tissues were grouped as follows: 0/15 (0%) well-, 7/33 (21%) moderate-, and 5/16 (31%) poor-differentiated (Figs. 10e–g, 10h). We found a statistically significant increase for phospho-CAV1 staining toward histologically poor differentiation (p<0.05). Furthermore serial sections of HCC tissues from the same patient showed co-expression of phospho-Met and phospho-CAV1 (Fig. 10i). Trend in χ^2^-test showed a statistically significant increase towards reduced histologic differentiation (p<0.05) (Fig. 10i). There was no correlation between staining patterns or intensity and none of the clinicopathological data (p>0.05).

## Discussion

Despite extensive efforts by many researchers, treatment with several different agents for HCC has been ineffective. Current use of sorafenib, a multikinase inhibitor, in treatment of advanced HCC, have focused research efforts on several key molecular pathways implicated in the pathogenesis of HCC [34],[35].

Others and we reported that the overexpression of c-Met in HCC is linked to an unfavorable clinicopathological status, including a low degree of differentiation, vascular invasion and metastasis [5],[6],[8]–[10]. Down regulation of c-Met expression by RNA interference or blockage of c-Met activation by specific inhibitors decreases invasion of HCC cells [5],[31],[35]. Disruption of CAV1 in invasive HCC cells suppresses *in vitro* migration and invasion [26]–[28] and also *in vivo* tumorigenicity and metastasis [28],[29]. In this study we tested the potential crosstalk between c-Met and CAV1 in HCC, and determined a co-localization between c-Met and CAV1 in HCC tissues, whereas there were no co-localization in cirrhotic liver tissues (Fig. S3). In addition, we observed that both c-Met and CAV1 expression is higher in poor-differentiated, highly motile and invasive HCC cell lines compared to well-differentiated ones. Involvement of c-Met and CAV1 overexpression in invasion, metastasis and differentiation of HCC cells suggested that their cooperativitiy might have a particular function during hepatocarcinogenesis.

Although there are many studies reporting the overexpression of either c-Met or CAV1 in HCC, the activation status of c-Met and CAV1 remains unclear. Our IHC analysis revealed a lack of c-Met and CAV1 activation in normal hepatocytes. However, activating phosphorylation levels of both CAV1 and c-Met as well as their co-localizations were increased in primary cirrhotic liver, and HCC tissues. To our knowledge, this is the first report that showed the activation levels and co-localization of c-Met and CAV1 in HCC tissues. Furthermore, increased activation of both phospho-Met and phospho-CAV1 in poorly-differentiated HCC tissues supported the role of this cooperation in the progression of HCC.

Studies performed in different cancer models revealed that insulin treatment, c-Src kinase activation and integrin-mediated mechanotransduction cause tyrosine phosphorylation of CAV1 on tyrosine 14 (Y14), which appears to be essential for CAV1-driven cell migration [36]–[39]. However, nothing is known about the mechanism(s) of CAV1 activation in HCC. In this study, our findings provide mechanistic evidence for CAV1 activation in HCC. Specifically, phosphorylation of CAV1 on Tyrosine 14 induced by HGF and blockade of the CAV1 phosphorylation by c-Met inhibitor was clearly proved c-Met-mediated CAV1 activation in HCC. Moreover, our data showed stimulatory effect of CAV1 on c-Met signaling, as overexpression of CAV1 accelerated HGF-induced c-Met, and silencing of CAV1 reduced activation of c-Met. These data indicated bidirectional crosstalk between CAV1 and c-Met signaling. HGF induced c-Met/CAV1 complex formation and enhanced co-localization of phosphorylated-c-Met with phosphorylated-CAV1, predominantly accumulated in the perinuclear region of cells. This data is consistent with the data reported by Cho et al [2012] that HGF induces the internalization of c-Met, which is co-localized with endocytic machinery members including CAV1 in 239E human embryonic kidney cell line [40]. They demonstrated that, CAV1 has a mediator function for c-Met trafficking to the perinuclear region towards signal promotion and inhibition of the association between endocytic machinery and c-Met inhibits HGF induced phosphorylation of MAPK and consequently reduces scattering and migration [40]. Accumulation of CAV1 and activated c-Met in the perinuclear region suggested ligand-mediated receptor internalization. Receptor mediated endocytosis occurs via ligand binding, either to increase signaling by associating internalized receptor with signaling targets localized to endosomes or to decrease signaling by sorting receptors to the lysosome for degradation [41]. Since lipid rafts and CAV1 are also important for the regulation of receptor degradation by a non-classical endocytic pathway [42], we evaluated the c-Met expression level after HGF treatment. HGF treatment had no effect on the c-Met expression level, whereas CAV1-overexpressing cells had a higher c-Met level compared to control cells. Under these experimental conditions we did not observe any alteration in the ubiquitination of c-Met after HGF stimulation (data not shown). These results and sustained activation of c-Met in CAV1-overexpressed HuH-7 clones supported that CAV1 participated in endosomal trafficking for sustained signal output; rather than sorted c-Met for proteosomal/lysosomal degradation (Fig. S2).

It has been reported that, endocytic trafficking of c-Met induces sustained p44/42-MAPK activation and its translocation to focal adhesion site and increases cell migration [43]. In this regards, we evaluated activation statue of p44/42-MAPK (Erk1/2) as an indicator of c-Met activation. As expected, parallel to c-Met activation, p44/42-MAPK activation was observed in CAV1-overexpressing cells. Furthermore, HGF-mediated p44/42-MAPK activation was abolished by SU11274 and CAV1-siRNA due to the downregulation of c-Met activation.

We evaluated the impact of CAV1 on c-Met signaling and its functional impotance by examining c-Met driven cellular activities. In accordance with the higher degree of c-Met activation upon stimulation with HGF; higher migration and invasion rates were determined for CAV1-overexpressing cells under HGF stimulation. The overexpression of CAV1 drastically increased branching-morphogenesis. On the other hand CAV1 silencing impaired HGF-mediated branching-morphogenesis and reduced the migratory and invasive potential of SNU-449 cells. Although CAV1 has been reported to require for vascular morphogenesis [44], to our knowledge there is no study representing the role of CAV1 in branching-morphogenesis in HCC. Therefore, our data indicated that CAV1 is required for maintaining HGF/c-Met-mediated aggressive phenotype in HCC.

**Figure 11:**
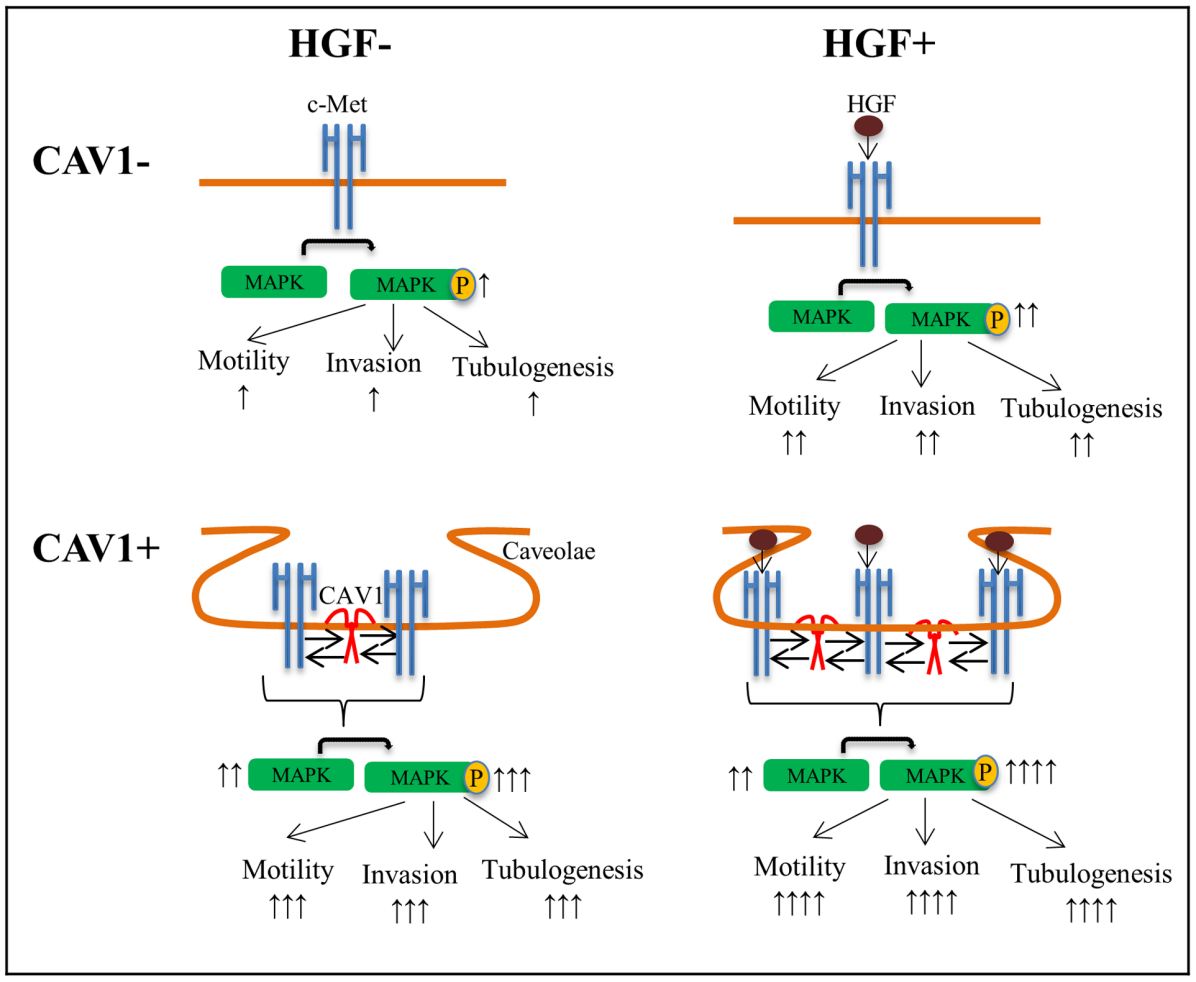
Model for Regulation of c-Met Receptor signaling by CAV1 in HCC cells. **A.** In the absence of CAV1 c-Met activation is in stationary phase **B.** CAV1 expression induces c-Met and MAPK activation and, c-Met driven motility, invasion and morphogenesis. **C.** HGF induced c-Met activation elevates migration, invasion and branching-morphogenesis in the absence of CAV1. **D.** HGF treatment induces the highest motility, invasion and morphogenesis in CAV1 expressing cells.

Taken together in this study we described a reciprocal activating crosstalk between c-Met and CAV1 that modulates motility, invasion and branching-morphogenesis of HCC cells. These conclusions are supported by the following findings: i) c-Met co-localized with CAV1 and this association increased by HGF; ii) HGF induced the activation of c-Met and CAV1; iii) association of activated c-Met with CAV1 resulted in increased migration, invasion and branching morphogenesis; iv) co-existence and colocalization of activated c-Met and CAV1 in HCC tissues (Fig. 11).

Conclusively, our results suggest that the bidirectional interactions between c-Met and CAV1 reciprocally and positively regulate each other's activity and have a critical influence on the outcome of c-Met signaling pathway in HCC. We propose CAV1 as a potential factor that triggers c-Met signal transduction and contributes to the initiation and progression of HCC. Therefore, targeting the cross-talk between c-Met receptor tyrosine kinase and CAV1 might be an important approach for the treatment of HCC.

## Supporting Information

Figure S1 **HGF induced association of CAV1 with c-Met.** **A.** Serum starved Mahlavu cells were exposed to HGF for 15, 30 and 60 min. Whole cell lysates were immunoprecipitated with anti-c-Met antibody, resolved by SDS-PAGE and immunoblotted with antibody to CAV1 and c-Met. Anti-c-Met antibody was probed to the membrane as a loading control. There was no detectable c-Met and CAV1 band in immunoprecipitates prepared with IgG as an IP-control. **B.** Mahlavu cells were analyzed for c-Met phosphorylation and expression by WB analysis by using anti-phospho-Met and anti-c-Met antibodies with or without HGF stimulation. Immunoblot analysis showed a gradual increase in c-Met phosphorylation in response to 15, 30, 60 min HGF induction. The expression level of CAV1 was also determined by WB under the described conditions. Calnexin was used as a loading control.
(TIF)

Figure S2 **HGF mediated c-Met and p44/42-MAPK activation.** WB showing the activation of the indicated proteins in HuH-7 and HuH-7-pCAV1 cells, overnight starved and treated with or without HGF at different time points. Calnexin was used as a loading control.
(TIF)

Figure S3 **Immunofluorescence analysis of co-localization between c-Met and CAV1.** IF microscopy acquisition of three fluorescent signals: c-Met (green Alexa 488) (a, d), CAV1 (red Alexa 555) (b, e) and DAPI (blue nuclear staining). Co-localization of c-Met and CAV1 was shown in yellow (c, f) (Bar = 200 µm).
(TIF)

Table S1 **Clinicopathological characteristics and phospho-Met and phospho-CAV1 immunohistochemical staining of tumors from primary HCC patients.**
(DOCX)
