# Supplementary figures and images for: Reciprocal Activating Crosstalk between c-Met and Caveolin 1 Promotes Invasive Phenotype in Hepatocellular Carcinoma
Source: PLoS One. 2014 Aug 22;9(8):e105278. doi: 10.1371/journal.pone.0105278 (PMC4141763; doi:10.1371/journal.pone.0105278)

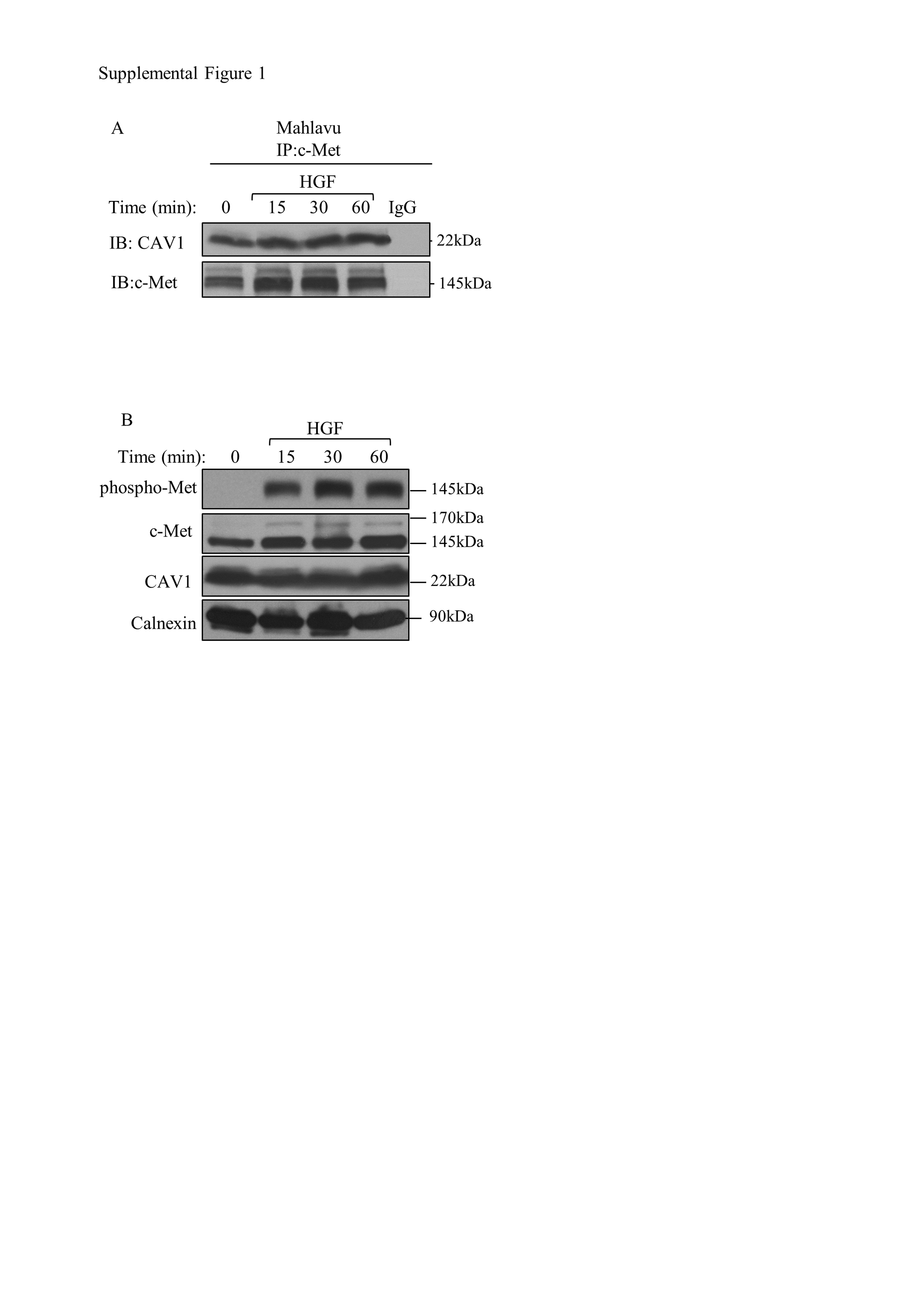

Supplement: Figure S1 — HGF induced association of CAV1 with c-Met. A. Serum starved Mahlavu cells were exposed to HGF for 15, 30 and 60 min. Whole cell lysates were immunoprecipitated with anti-c-Met antibody, resolved by SDS-PAGE and immunoblotted with antibody to CAV1 and c-Met. Anti-c-Met antibody was probed to the membrane as a loading control. There was no detectable c-Met and CAV1 band in immunoprecipitates prepared with IgG as an IP-control. B. Mahlavu cells were analyzed for c-Met phosphorylation and expression by WB analysis by using anti-phospho-Met and anti-c-Met antibodies with or without HGF stimulation. Immunoblot analysis showed a gradual increase in c-Met phosphorylation in response to 15, 30, 60 min HGF induction. The expression level of CAV1 was also determined by WB under the described conditions. Calnexin was used as a loading control. (TIF) [file pone.0105278.s001.tif]

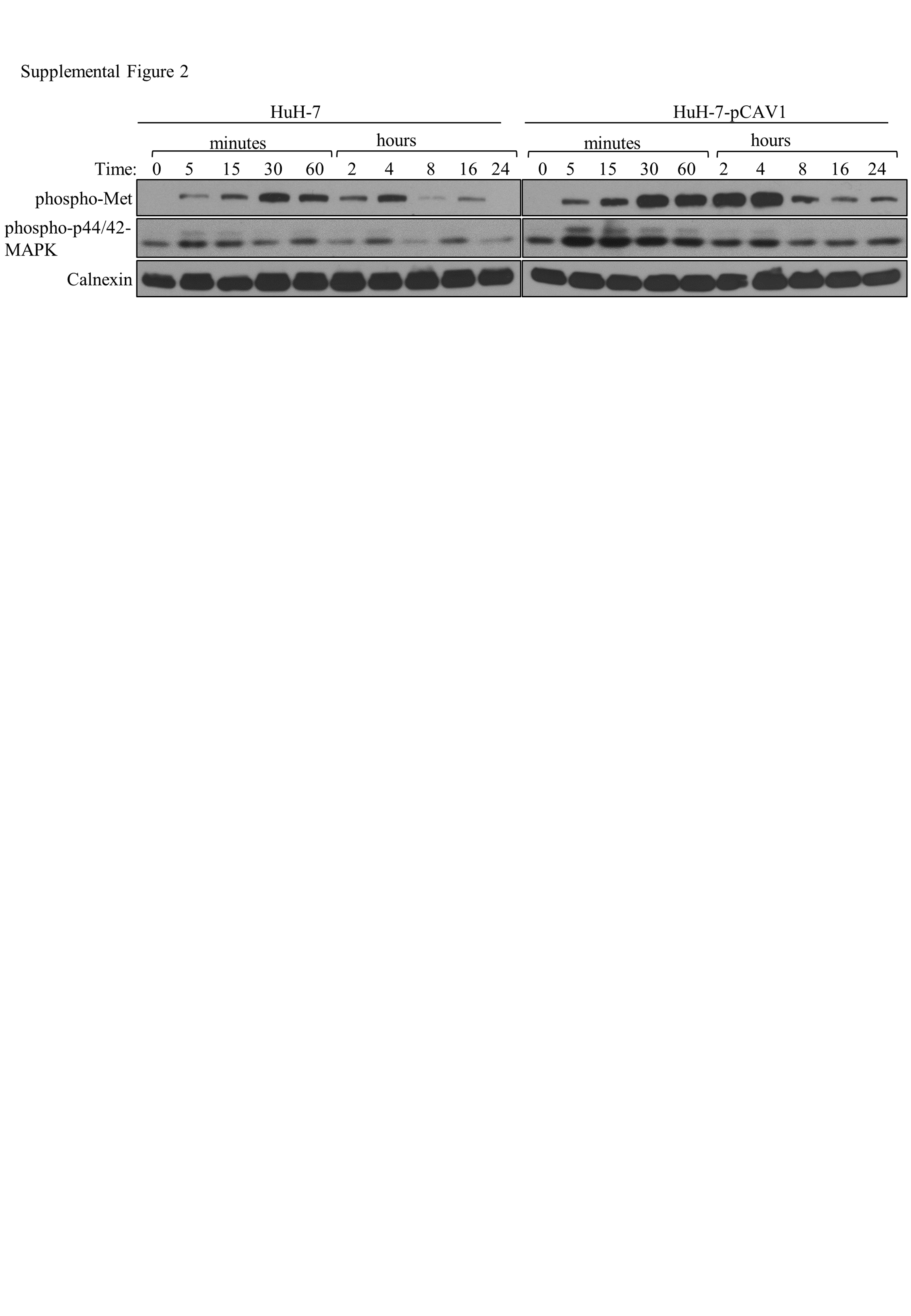

Supplement: Figure S2 — HGF mediated c-Met and p44/42-MAPK activation. WB showing the activation of the indicated proteins in HuH-7 and HuH-7-pCAV1 cells, overnight starved and treated with or without HGF at different time points. Calnexin was used as a loading control. (TIF) [file pone.0105278.s002.tif]

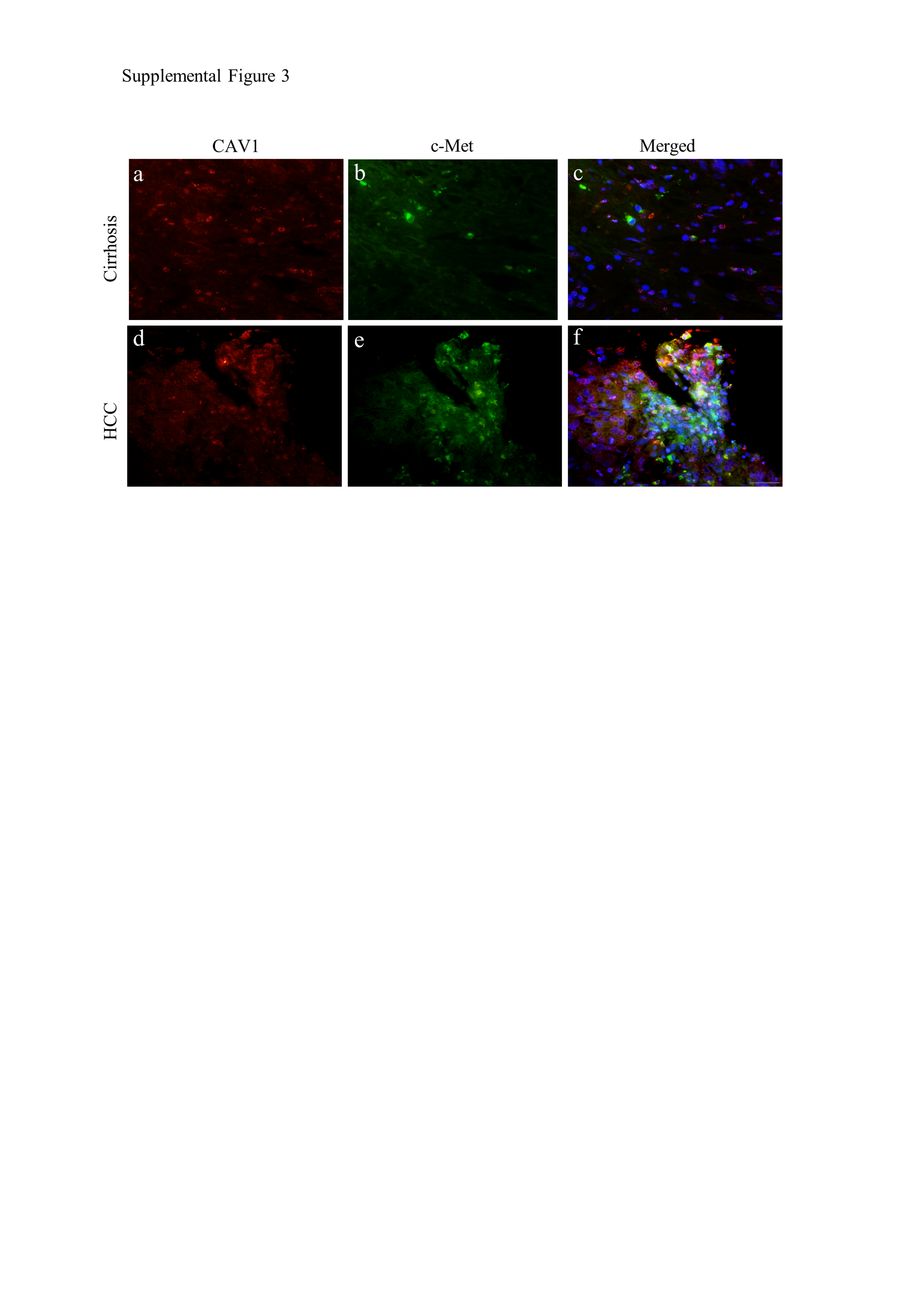

Supplement: Figure S3 — Immunofluorescence analysis of co-localization between c-Met and CAV1. IF microscopy acquisition of three fluorescent signals: c-Met (green Alexa 488) (a, d), CAV1 (red Alexa 555) (b, e) and DAPI (blue nuclear staining). Co-localization of c-Met and CAV1 was shown in yellow (c, f) (Bar = 200 µm). (TIF) [file pone.0105278.s003.tif]
