# Supplementary material for: Reciprocal Activating Crosstalk between c-Met and Caveolin 1 Promotes Invasive Phenotype in Hepatocellular Carcinoma
Source: PLoS One. 2014 Aug 22;9(8):e105278. doi: 10.1371/journal.pone.0105278 (PMC4141763; doi:10.1371/journal.pone.0105278)
Supplement: Table S1 — Clinicopathological characteristics and phospho-Met and phospho-CAV1 immunohistochemical staining of tumors from primary HCC patients. (DOCX) [file pone.0105278.s004.docx]

**TABLE**

**Table S1:** Clinicopathological characteristics and phospho-Met and phospho-CAV1 immunohistochemical staining of tumors from primary HCC patients.

| **Parameters** | variable | n(%) |
| --- | --- | --- |
| Gender | Female | 17 |
|  | Male | 83 |
| Tumor size (cm) | ≤5 | 72 |
|  | >5 | 28 |
| Nodule number (n) | ≤3 | 78 |
|  | >3 | 22 |
| Etiology | Viral | 81 |
|  | Alcohol | 3 |
|  | Others | 17 |
| Venous invasion | Present | 30 |
|  | Absent | 70 |
| Tumor grade | Well | 25 |
|  | Moderate | 56 |
|  | Poor | 19 |
